# Augmented reality for sentinel lymph node biopsy

**DOI:** 10.1007/s11548-023-03014-w

**Published:** 2023-09-25

**Authors:** Peter A. von Niederhäusern, Carlo Seppi, Robin Sandkühler, Guillaume Nicolas, Stephan K. Haerle, Philippe C. Cattin

**Affiliations:** 1 Department of Biomedical Engineering, University of Basel, Allschwil, Switzerland; 2 Nuclear Medicine Clinic, University Hospital Basel, Basel, Switzerland; 3 Hirslanden Clinic Lucerne, Lucerne, Switzerland

**Keywords:** Sentinel lymph node biopsy, SNB, Nuclear medicine, Gamma camera, Gamma detector, Augmented reality, AR, Inverse problem

## Abstract

**Introduction:**

Sentinel lymph node biopsy for oral and oropharyngeal squamous cell carcinoma is a well-established staging method. One variation is to inject a radioactive tracer near the primary tumor of the patient. After a few minutes, audio feedback from an external hand-held $\gamma$-detection probe can monitor the uptake into the lymphatic system. Such probes place a high cognitive load on the surgeon during the biopsy, as they require the simultaneous use of both hands and the skills necessary to correlate the audio signal with the location of tracer accumulation in the lymph nodes. Therefore, an augmented reality (AR) approach to directly visualize and thus discriminate nearby lymph nodes would greatly reduce the surgeons’ cognitive load.

**Materials and methods:**

We present a proof of concept of an AR approach for sentinel lymph node biopsy by *ex vivo* experiments. The 3D position of the radioactive $\gamma$-sources is reconstructed from a single $\gamma$-image, acquired by a stationary table-attached multi-pinhole $\gamma$-detector. The position of the sources is then visualized using Microsoft’s HoloLens. We further investigate the performance of our SLNF algorithm for a single source, two sources, and two sources with a hot background.

**Results:**

In our *ex vivo* experiments, a single $\gamma$-source and its AR representation show good correlation with known locations, with a maximum error of 4.47 mm. The SLNF algorithm performs well when only one source is reconstructed, with a maximum error of 7.77 mm. For the more challenging case to reconstruct two sources, the errors vary between 2.23 mm and 75.92 mm.

**Conclusion:**

This proof of concept shows promising results in reconstructing and displaying one $\gamma$-source. Two simultaneously recorded sources are more challenging and require further algorithmic optimization.

## Introduction

The currently established protocol for sentinel lymph node biopsy (SNB) for oropharyngeal cancer treatment involves monitoring the uptake of a circum-tumor (*e.g.*, in the tongue) injected radioactive ^99 m^Tc-labeled tracer [1]. A hand-held $\gamma$-detection probe (HGDP) is used to measure the uptake. The highest uptake is likely to be observed in sentinel lymph nodes (SLN), downstream of the tumor, and at the injection site. Post-extraction analysis of these SLNs then allows the assessment of the spreading. If no malignant cells are found (*i.e.*, clinically negative neck, cN0), the tumor has not yet begun to spread. Otherwise, elective neck dissection (END) is performed where parts of the lymphatics of the neck are removed in the hope of containing occult metastases. An indication of a cN0 shows a good prognosis for 70 % of such cases, and the need for an END is thus unjustified [2]. Many centers conduct neck dissection for all their patients, therefore overtreating most of them.

The HGDP approach converts the activity of the tracer accumulation into audio feedback. Relying on such feedback requires spatial awareness on the part of the operator to correlate it with the actual position of the detected hot spots, resulting in a high cognitive load. Operating the HGDP interrupts the excision process and adds complexity to the workflow. SPECT/CT can help localize the SLN and improve surgical planning but does not alleviate the need for real-time intraoperative localization. The use of augmented reality (AR) for navigation could reduce the surgeon’s workload and assist them in performing accurate SLNs, potentially reducing the risk of missing relevant lymph nodes and avoiding overtreatment.

**Fig. 1:**
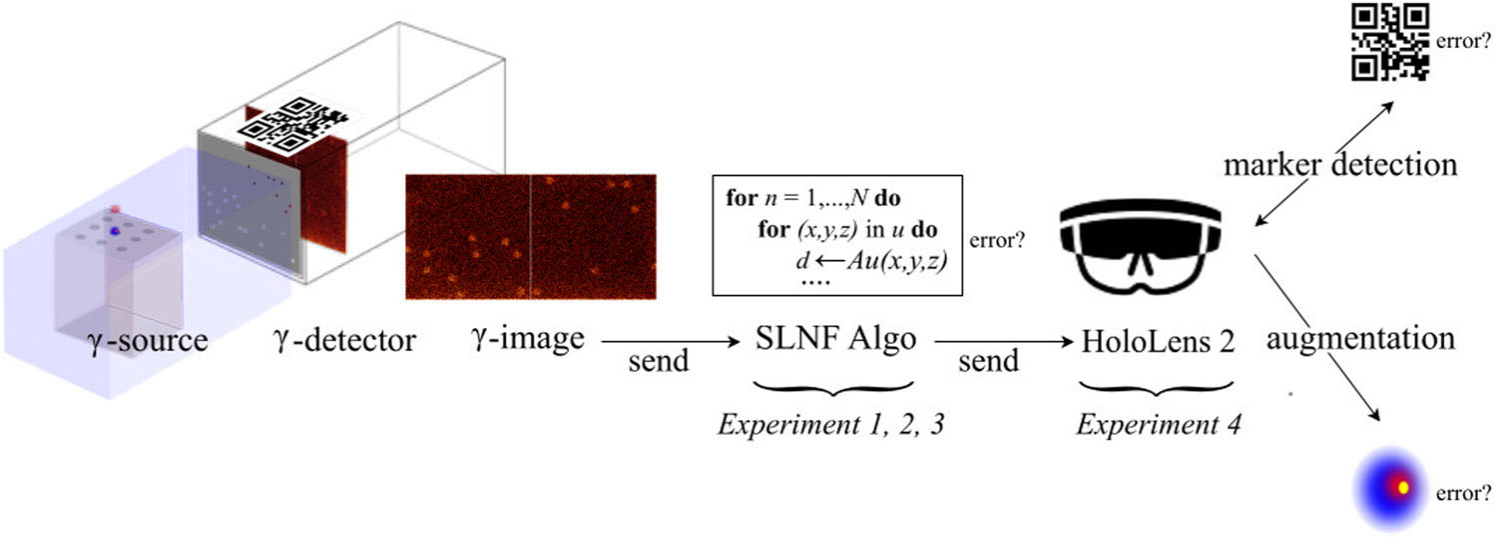
Pipeline: overview of our method. From the $\gamma$-source to the AR headset

AR in surgery is gaining traction, *e.g.*, in spinal fusion surgery and orthopedics [3], or in assessing robot-assisted approaches and procedures in minimally invasive surgery [4]. A medical device combining a classical HGDP with AR is commercially available.^1^ This freehand SPECT device differs from our envisioned approach, as it requires, for example, an optical outside-in tracking system. Further, the augmentation is not displayed in the surgeon’s field of view (FoV). Our integrated approach is based on a pipeline (Fig. 1): the influence of errors (*cf. *Fig. 1, question marks), *e.g.*, filter settings of the $\gamma$-detector, reconstruction inaccuracies, and pose estimation imprecision, were experimentally evaluated. In the first stage, the detector registers the $\gamma$-source activity. In subsequent stages, the image is sent to the reconstruction algorithm, and the computed position of the source is transferred wirelessly to the AR headset and displayed accordingly. In this study, the first three experiments assess the reconstruction algorithm under different conditions, and the fourth experiment examines the actual AR quality of the reconstruction.

**Fig. 2:**
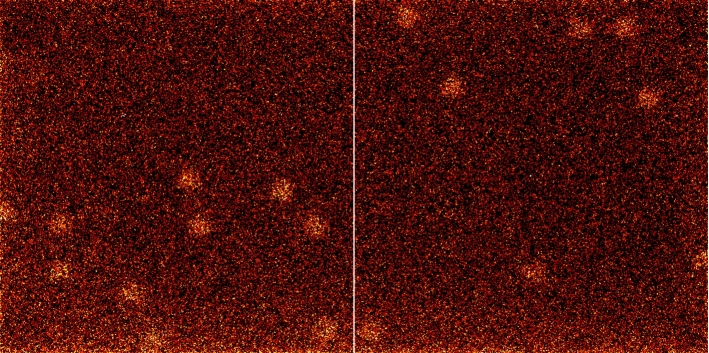
$\gamma$-Image of a single $\gamma$-source. Circular $\gamma$-photon accumulations are clearly discernible. These are produced by the multi-pinhole collimator pattern. The vertical line is a sensor artifact

## Materials and methods

### Materials

We used a 750 $\upmu$m-thick cadmium telluride (CdTe) crystal $\gamma$-detector with a hybrid photon counting (HPC) CMOS chip [5]. Its pixel size is 75 $\upmu$m^2^, with an image resolution of 1030 $\times$ 514 pixels, and quantum efficiency of 10 % at 140.5 keV for ^99m^Tc [6]. The device supports two thresholds in the range of [9 keV, 70 keV] to filter out $\gamma$-rays of a specific keV regime. In a preprocessing step, all the images were binned (summing pixel neighborhoods) to 515 $\times$ 257 pixels to be computationally more efficient (Fig. 2). Our industrial partner DECTRIS Ltd, Baden, Switzerland, provided the device. A custom 3D printed tungsten (W) collimator was placed in front of the sensor [7]. This element has a mass attenuation coefficient^2^ of 1.58 ${\textrm{cm}}^{2}\,{\textrm{g}}^{-1}$ at 140.5 keV.

The size and weight of the collimators are often not considered to be limiting factors, given their application scenario in a fixed setting, *e.g.*, embedded into a stationary SPECT/CT device for preoperative imaging. This, however, changes for radio-guided surgery where, due to limited space in the operation room, only a small collimator is applicable. Hence, a trade-off between the aforementioned characteristics has to be made. The overall size of the collimator/detector is d $\times$ h $\times$ w: 280 $\times$ 90 $\times$ 110 mm^3^. The overall weight of the $\gamma$-detector and collimator is 4005 g. As our compact collimator consists of relatively thin walls, their shielding properties are limited and facilitate the unwanted penetration of $\gamma$-photons, *i.e.*, 5 % to 10 %, blurring the image. We used a multi-pinhole collimator, inspired by the collimator proposed by [8] (see Appendix A.2 for more details). The pinholes have a diameter of 1 mm, a thickness of 1 mm, and a FoV of 90 ${}^{\circ }$. Microsoft’s HoloLens 2 (HL2) has the ability to do inside-out tracking [9]. With its spatial mapping functionality, based on integrated sensors (*i.e.*, IMUs, infrared-based depth sensors) and the Mixed Reality Toolkit (MRTK), no additional tracking hardware is needed to align real and virtual objects [10, 11]. The optics of the waveguide display enable a FoV of approximately 53 ${}^{\circ }$. To simulate SLNs, we used ^99m^Tc sources for the experiments. These ^99m^Tc sources were filled into 0.5 mL vials with activities of 5 MBq, 15 MBq, 30 MBq, and 60 MBq, respectively. In addition, we used a small amount of tracer with an activity of 120 MBq for the cellulose/lignin matrix of the wooden block to emit constant radiation to simulate a hot background. These activities are high compared to tracer-enriched tissue. However, an increased exposure time for *in vivo* experiments with lower activity can compensate for this.

### Methods

#### Reconstruction

**Fig. 3:**
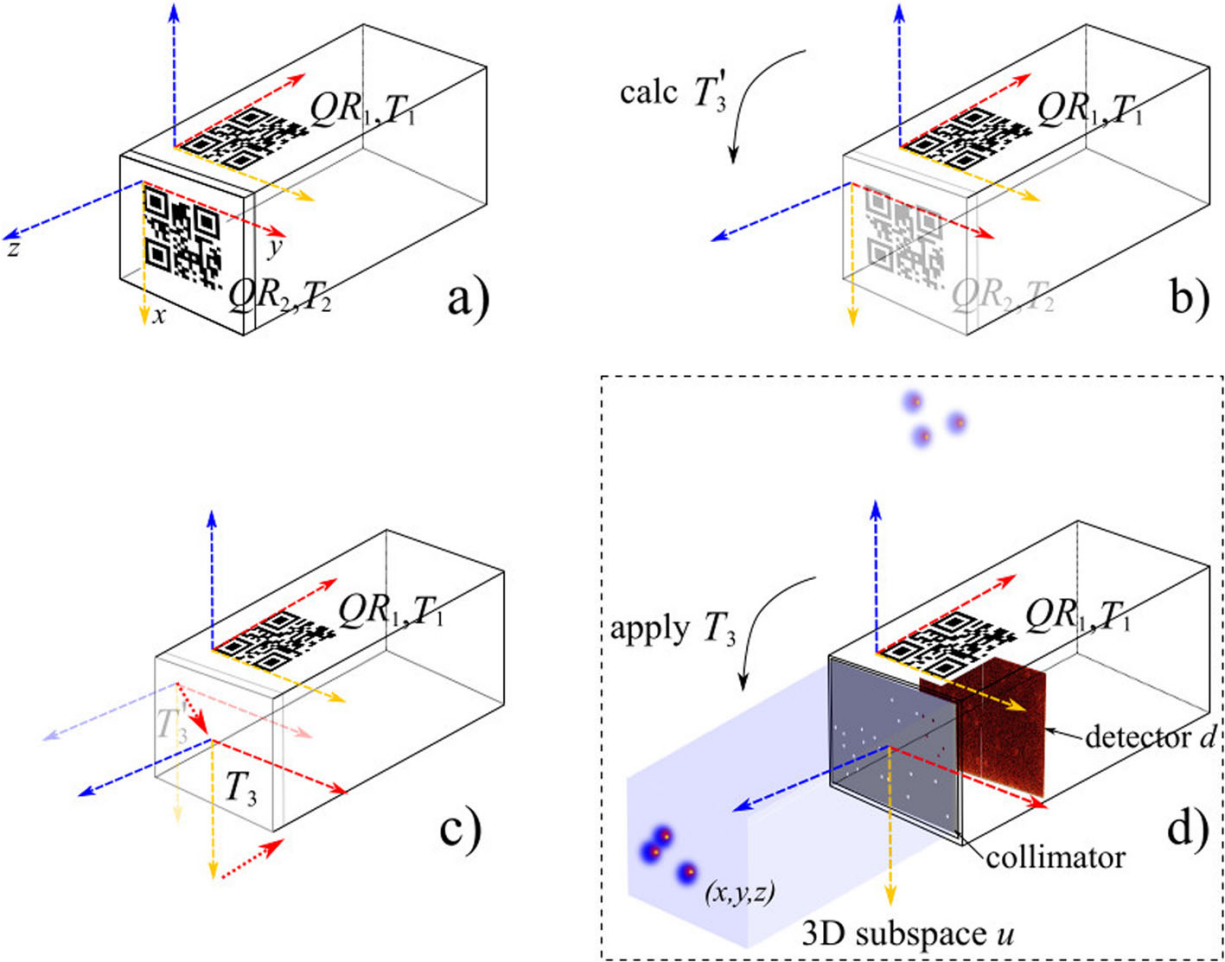
Calibration: **a** Detecting the QR marker $QR_1$ to determine the *liminal* space $T_1$ (scene anchoring), followed by detecting $QR_2$ to yield $T_2$. **b** $T_3'$ is the mapping from $T_1$ to $T_2$. **c** Adapting $T_3'$ to yield $T_3$ (dotted arrows). **d** Augmentation (boxed): applying $T_3$ to display the augmentation in the correct location (*i.e.*, source (*x*, *y*, *z*)). $QR_2$ is only needed during calibration

We reconstructed the ^99m^Tc distribution *u* in a 3D subspace using a single detector image (Fig. 3d), as described in [8, 12]. We simplified our model by assuming that the photons travel in a straight line from the source through the pinholes to the detector. Therefore, the relationship between the 3D subspace $u\in {\mathbb {R}}^M_{\ge 0}$ and the 2D detector image $d^{proj}\in {\mathbb {R}}^N_{\ge 0}$ can be described by a linear mapping operator $A\in \{0,1\}^{N\times M}$, with

1 $$\begin{aligned} Au=d^{proj}. \end{aligned}$$

We used $d^{proj}$ and *u* as vectors, so we can describe their relationship as a matrix–vector multiplication (Eq. 1). The entry $A_{ij}$ of the matrix *A* is 1, if a straight line through the pinhole from the voxel $u_i$ to the pixel $d^{proj}_j$ exists, and 0 otherwise. *N* is the number of pixels from the detector image and *M* is the number of the voxels from the 3D subspace. The sentinel lymph node fingerprinting (SLNF) algorithm [8] finds the position of the ^99m^Tc source. SLNF (*cf. *Algorithm 1) compares the measured detector image $d^{obs}$ with the projected detector image $d^{proj}$. Note that for a different position of the simulated ^99m^Tc source *u*, the projected detector image $d^{proj}$ changes. For simplicity, the ^99m^Tc source *u* is approximated as a point source in the projection (*u* has exactly one entry set to 1, and all others are set to 0), such that *u* corresponds to a (*x*, *y*, *z*) resulting in a unique $d^{proj}$. We use the SLNF algorithm to find the (*x*, *y*, *z*) position of the ^99m^Tc source; hence, we find (*x*, *y*, *z*) s.t. $d^{proj}$ maximizes $C(d^{proj},d^{obs})$:

2 $$\begin{aligned} \mathop {\mathrm {arg\,max}}\limits _{d^{proj}} C(d^{proj},d^{obs})= \mathop {\mathrm {arg\,max}}\limits _{d^{proj}} \sum _{i} \left( d^{proj}_{i}\cdot d^{obs}_{i} \right) , \end{aligned}$$

where $d^{obs}$ is the observed detector image from the experiment. We have made a small adaptation to the SLNF algorithm described in [8]. Namely, we assume that the number of sources is known. Note that in line 4 of Algorithm 1, the indices of $d_i^{obs}$ are set to zero where $d^{proj}_{i} > 0$, so that the information of the source found is removed, and the information of the next source is dominant. See [8] for a more detailed explanation.

**Algorithm 1:**
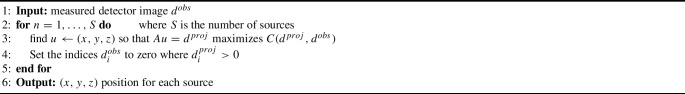
SLNF: finding the position of the radioactive ^99m^Tc source.

To decrease the runtime of the algorithm, we reduced the size of the matrix *A* and the detector image $d^{obs}$. The matrix occupies a subspace volume (*x*, *y*, *z*) of 60 $\times$ 100 $\times$ 200 sampling points with a spatial sampling distance of 2 mm in each dimension. The detector image size is 515 $\times$ 257 sampling points, in accordance with the binned image size (Sect. “Materials and methods”). Since the *z*-range is limited to 200 mm, we only measure and reconstruct activity within this depth range. The anatomy of the neck and the lymphatics are usually covered within this range.

**Fig. 4:**
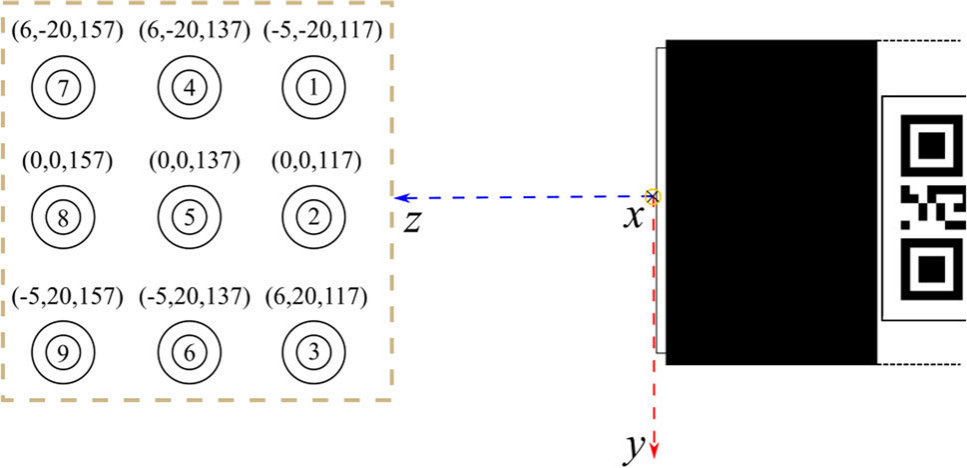
Setup of measurement space (Experiments 1, 2, and 3), view from above. Wooden block for the vials, their source positions (tuples (*x*, *y*, *z*), [ mm]) with the origin in the middle of the collimator plate (coordinate system with axes *x*, *y*, *z* drawn), and their associated index ${\textcircled {1}}$ to ${\textcircled {9}}$; the $\gamma$-detector is to the right (schematics)

*Assessment of the source positions*  An optimal design algorithm was introduced in [13], which the authors in [8] applied to find a pinhole pattern for the collimator. In Appendix A, we describe the transformation of the optimal design algorithm so that we could assess the ability to reconstruct different positions using our collimator. To compare the nine positions given in Fig. 4 and assess which positions will be challenging to reconstruct, we assumed that each is a point source. That being the case, all values of the subspace *u* are 0 except for one. We used solely the column of mapping operator *A* (see Eq. 1) corresponding with the nonzero value of *u*, which is the projection of *u* onto the detector. Thus, we can write for the sources $i=1,\dots ,9$ the vector $d^{(i)}$ corresponding to the detector image, which is one of the columns of the matrix *A*. Using the transformation of the optimal design algorithm described in Appendix A.3, the performance of the SLNF algorithm can be assessed for each source $i=1,\dots ,9$ with

3 $$\begin{aligned} \tilde{{\mathcal {V}}}(i) = \text {trace}\left( d^{(i)}\left( d^{(i)T}d^{(i)}\right) ^{-2}d^{(i)T}\right) . \end{aligned}$$

#### Calibration and augmentation

A calibration step establishes a correct transformation between the different coordinate systems or frames (Figs.  3, (a) - (c)). We start by letting the HL2 register the marker $QR_1$ attached on top of the $\gamma$-detector, by leveraging the MRTK [11]. This initial detection yields the *liminal* space from which all further augmentation is computed. Therefore, detecting and tracking the marker $QR_1$ by the HL2’s cameras is the anchor for scene augmentation. We also detect the marker $QR_2$ that represents the collimator. This collimator-attached marker is only needed during calibration. As long as the relationship between the detector’s marker $QR_1$ and the collimator, attached to the front, is not changed, no re-calibration is necessary.

The obtained calibration parameters are stored and reused for the actual augmentation, in conjunction with the current world position of $T_1$ from $QR_1$, and the computed dependent transform $T_3$ (Fig. 3d).

#### Experiments

We investigated the performance of the SLNF-based 3D reconstruction of $\gamma$-sources and the quality of their 2D AR representation. For each experiment, we present the setup and the data. The measurement space with the ground truth location of the sources and their indices for the result tables is shown in Fig. 4. Experiments 1, 2, and 3 evaluate the errors introduced by the SLNF reconstruction algorithm, where the complexity increases with the scene setup, *i.e.*, one source, two simultaneous sources, and two simultaneous sources with a hot background. We initially tested with two thresholds set to 9 keV and 49 keV on the $\gamma$-detector. We then only used the lower threshold because there was no significant difference in the results. Experiment 4 evaluates the augmentation errors, *i.e.*, the reconstruction errors and the pose estimation errors, to obtain the *overall performance* of our approach. Each source position was measured 10 times, with an exposure time of 8 s/sample. For each experiment, we report the median and the 3rd quartile $Q_3$ of the error $\varepsilon$ of the sequence; $Q_3$ provides additional insight into the resulting error distribution.

**Fig. 5:**
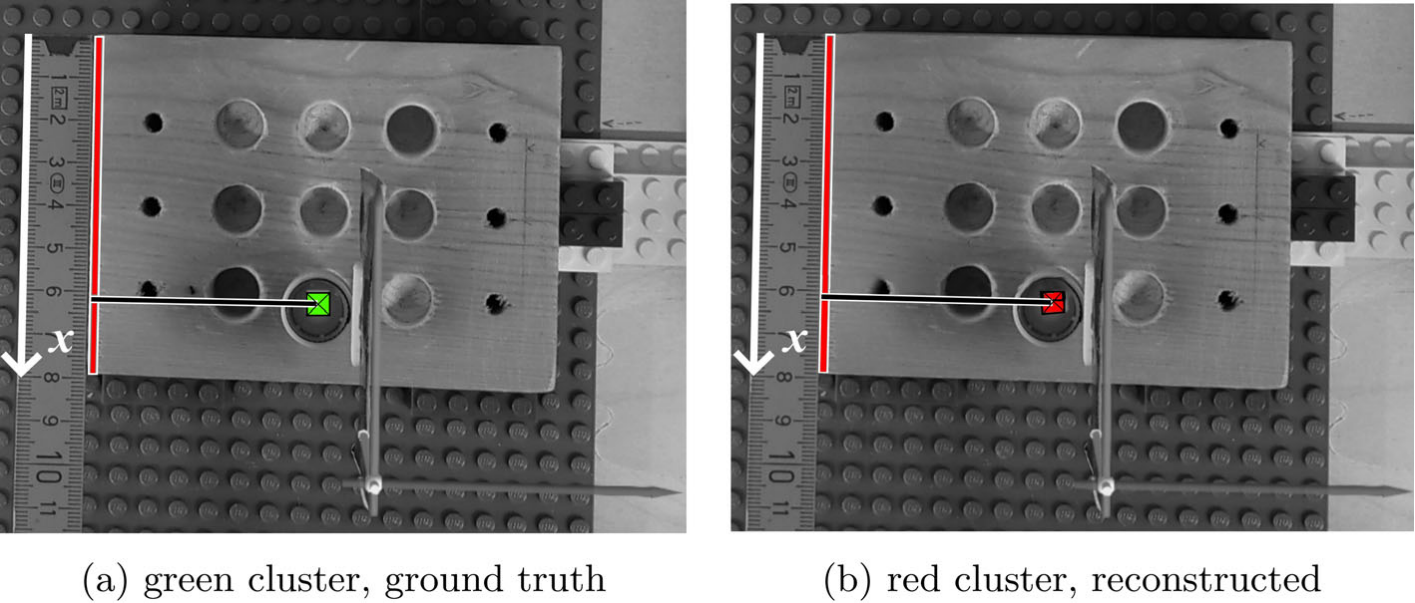
Assessment of the *x*-coordinate. **a** The center of the ground truth cluster is anchored on the red line with its origin in the top left of the wooden block (white arrow). **b** The red cluster is compared against the same reference line. The two clusters have a projected distance of approximately 61 mm along the *x*-axis from the reference point in the top left

**Fig. 6:**
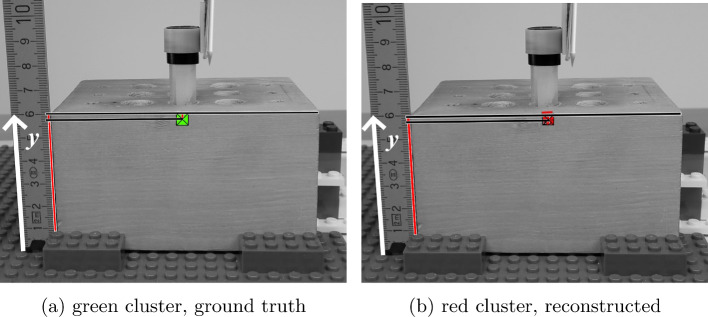
Assessment of the *y*-coordinate. **a** The center of the ground truth cluster is anchored on the black reference line of the wooden block top edge (white arrow). **b** The red cluster is compared against the same reference line. The two clusters have a projected distance of approximately 58 mm along the *y*-axis to the reference line

*Experiment 1, reconstruction of one source*  In the first experiment, we used a single 0.5 mL ^99m^Tc source with activities of 5 MBq or 15 MBq that were placed at each of the positions of the wooden block (*cf. *source position indices Fig. 4).

*Experiment 2, reconstruction of two sources*  Simultaneously measuring two 0.5 mL ^99m^Tc sources with activities of 15 MBq at two different positions of the wooden block (*cf. *source position indices Fig. 4, Table 2).

*Experiment 3, reconstruction of two sources with a hot background*  Simultaneously measuring two 0.5 mL ^99m^Tc sources with activities of 30 MBq and 60 MBq, and with an induced hot background: A small amount of tracer with an activity of 120 MBq was put into the cellulose/lignin matrix of the wooden block to emit constant radiation (*cf. *source position indices Fig. 4, Table 3).

**Fig. 7:**
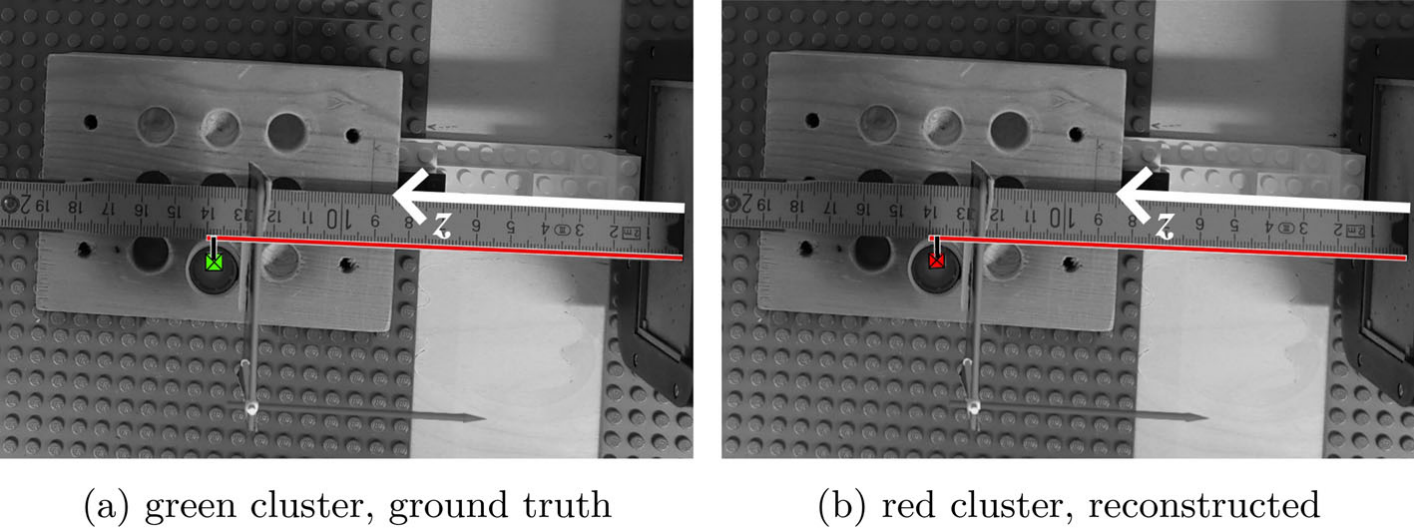
Assessment of the *z*-coordinate. **a** The center of the ground truth cluster is anchored on the red reference line with its origin at the collimator site (white arrow). **b** The red cluster is compared against the same reference line. The two clusters have a projected distance from the collimator of approximately 138 mm along the *z*-axis

**Table 1:** Experiment 1: a single 5 MBq source (upper row), and a single 15 MBq source (lower row)

	Position indices								
	1	2	3	4	5	6	7	8	9
Median ($\varepsilon$, [ mm]), 5 MBq	7.30	3.14	4.73	3.14	2.91	4.84	3.73	3.23	3.79
$Q_3$	12.48	3.46	7.5	4.79	4.67	6.55	4.47	5.96	4.58
Median ($\varepsilon$, [ mm]), 15 MBq	7.77	2.41	4.47	2.82	3.46	4.12	4.10	3.46	3.79
$Q_3$	11.88	3.30	5.86	3.46	4.89	4.89	4.47	3.46	4.58

The median and the 3rd quartile $Q_3$ of the error $\varepsilon$ of the SLNF-based reconstruction are shown for each position, compared to the ground truth. The error metric is the $\mathrm {{\ell }}^2$-norm

*Experiment 4, AR assessment*  The evaluation of the augmentation *accuracy* was done on the 2D projection of the *x*, *y*, *z* axes to compare the results from the SLNF-reconstructed source with the ground truth. As we did not use an external tracking system, a coordinate system different from the one used for Experiments 1, 2, and 3 was applied. The AR blended-in objects (clusters) were manually segmented. To reduce potential *drift* from the inside-out tracking of the HL2 [14] during measurement, we mounted an additional marker on top of the vial with the tracer; this marker is detected by the HL2 and served as ground truth (green cluster in Figs. 5, 6, and 7) for comparing the location of the SLNF-reconstructed source (red cluster in Figs. 5, 6, and 7) at that position. For each position, first-person view images from the perspective of the HL2 bearer were recorded for the main axes *x*, *y*, *z* (*cf. *Table 4, Figs. 5, 6, and 7). In each image, the ruler was used to give absolute values of the position of the cluster. The computation *speed* is mainly governed by the $\gamma$-detector settings, *i.e.*, exposure time and the requested number of images, and the runtime of the SLNF reconstruction algorithm. In our setup, we used an image series of 10 measurements/position with an exposure time of 8 s/measurement. The reconstruction algorithm took approx. 1.5 s to compute the position per measurement. Therefore, the update time for a new position was approx. 2 min. A scene redrawing for a computed position is done at a frame rate of approx. 16 fps (frames per second), given by the HL2’s render capabilities [14]. This is crucial for a good AR experience.

## Results

*Experiment 1, reconstruction of one source*  The overall median for the 5 MBq source is 3.73  mm with $Q_3$ at 4.79  mm. The overall median for the 15 MBq source is 3.79  mm with $Q_3$ at 4.58  mm. See Table 1 for detailed results.

*Experiment 2, reconstruction of two sources*  The lowest medians are for Positions 7, 9 (3.14  mm, 2.23  mm) with $Q_3$ at (4.47  mm, 2.80  mm). See Table 2 for detailed results.

*Experiment 3, reconstruction of two sources with a hot background*  The lowest medians are for Positions 2 (60), 3 (60), (4.47  mm, 6.32  mm) with $Q_3$ at (4.47  mm, 9.22  mm). See Table 3 for detailed results.

*Experiment 4, AR assessment*  We applied the $\mathrm {{\ell }^2}$-norm to calculate the error or accuracy between the ground truth and the reconstructed source. We proceeded accordingly for all nine positions of Table 4. The lowest error of 0.00 mm is at Position 6, and the highest error of 4.47 mm is at Position 3.

*Assessment of the source positions*  In Table 5, we compute the values of Eq. 3 for each of the nine sources and find that the highest value is at Position $i=1$ with 3e-04, which can explain the high error at Position 1 in Tables 1 and  2.

**Table 2:** Experiment 2: two simultaneous 15 MBq sources. For each position, the median and the 3rd quartile $Q_3$ of the error $\varepsilon$ of the SLNF-based reconstruction, compared to the ground truth, are shown

	Position indices					
	1	7	1	8	1	9
Median ($\varepsilon$, [ mm])	19.28	12.30	17.26	2.82	13.42	2.23
$Q_3$	25.58	16.12	18.24	4.47	15.82	2.23
	2	7	2	8	2	9
Median ($\varepsilon$, [ mm])	3.65	16.12	6.47	8.54	3.65	2.23
$Q_3$	5.86	16.12	13.31	13.68	4.47	2.23
	3	7	3	8	3	9
Median ($\varepsilon$, [ mm])	6.16	16.12	6.16	2.82	6.69	2.23
$Q_3$	6.32	16.12	6.32	2.82	8.00	3.99
	1	3	4	6	7	9
Median ($\varepsilon$, [ mm])	19.23	6.32	2.82	3.60	3.14	2.23
$Q_3$	21.70	6.32	4.06	7.92	4.47	2.80

The last position row shows two sources which are placed laterally with a distance of 40 mm along the *y*-axis. The error metric is the $\mathrm {\ell }^2$-norm

**Table 3:** Experiment 3: two simultaneous sources (30 MBq, 60 MBq), with hot background

	Position indices, activities (MBq)					
	2, (60)	3, (60)	2, (60)	3, (30)	2, (30)	3, (60)
Median ($\varepsilon$, [ mm])	4.47	6.32	3.14	67.49	6.32	6.32
$Q_3$	4.47	9.22	4.47	68.67	54.59	6.32
	3, (60)	5, (60)	3, (60)	5, (30)	3, (30)	5, (60)
Median ($\varepsilon$, [ mm])	6.32	24.59	74.35	20.85	6.32	21.00
$Q_3$	6.32	38.24	75.92	21.00	6.32	21.88

For each position, the median and the 3rd quartile $Q_3$ of the error $\varepsilon$ of the SLNF-based reconstruction, compared to the ground truth, are shown. In the top row, the two sources are placed laterally with a distance of 20 mm along the *y*-axis. In the bottom row, the two sources are placed along the diagonal, with a distance of 28.28 mm. The error metric is the $\mathrm {\ell }^2$-norm

**Table 4:** Experiment 4: exemplified for Position 6, we show the three axes *x*, *y*, *z* of the two clusters in their font (bold, ground truth, and italics, SLNF reconstruction), and the $\mathrm {{\ell }^2}$-norm [ mm] as the error

Position indices, (ground truth), (reconstruction): $\mathrm {{\ell }^2}$-norm [mm]		
7	4	1
(22, 78, 157), (22, 76, 157): 2.00	(19, 80, 137), (22, 80, 138): 3.16	(21, 62, 117), (21, 62, 121): 4.00
8	5	2
(41, 65, 157), (42, 67, 158): 2.45	(41, 67, 138), (41, 69, 139): 2.24	(41, 66, 117), (39, 68, 119): 3.46
9	6	3
(61, 57, 156), (61, 58, 159): 3.16	**(61, 58, 138)**, *(61, 58, 138)*: 0.00	(60, 67, 117), (56, 67, 119): 4.47

Coordinates are measured in the image domain, showcased in Figs. 5, 6, and 7, again for Position 6

**Table 5:** Scores of the optimal design algorithm. The specific multi-pinhole arrangement of this study introduces a systematic error for Position 1 (highest score)

	Positions								
i	1	2	3	4	5	6	7	8	9
$\tilde{{\mathcal {V}}}(i)$ score ($1\cdot \textrm{e}-$04)	3.00	2.64	2.70	2.77	2.56	2.82	2.74	2.52	2.64

This correlates with the reconstruction errors in Tables 1 and 2

## Discussion

*Experiment* 1 shows a stable SLNF reconstruction in the single-source, varying activity case. For both activities, the overall median is 3.76 mm, and the overall $Q_3$ is 4.73 mm (Table 5). For Position 1, the error distribution is highest due to the pattern design of the collimator (Sect. “Results”, Assessment of the source positions). The results of *Experiment* 2 and 3 show the limitations of the algorithm, with the highest medians and $Q_3$ of 19.28 mm & 25.58 mm, and 74.35 mm & 75.92 mm, respectively. The algorithm works iteratively: (1) computes the position of the strongest source, (2) masks it from the resulting intermediate result, (3) continues to try to find the remaining sources, even if none exist. This might lead to larger errors. However, the first source detected and reconstructed is in a similar error range as is shown in Experiment 1. In Experiment 2, with Positions 1 & 7, 1 & 8, and 1 & 9, the reconstruction of Position 1 shows a larger error than the other positions. Compared to all the other results, these reconstructions additionally suffer from the bias due to the pattern design of the collimator. The reconstruction errors for Positions 2 & 7, 2 & 8, and 2 & 9, are similar. The reconstruction for Positions 3 & 7, 3 & 8, and 3 & 9, reaches an error range comparable to Experiment 1. The error band narrows the further away the sources are placed from Position 1. In Table 2, the results are shown for Positions 1 & 3, 4 & 6, and 7 & 9, where two sources are laterally placed, *i.e.*, on the same row of the wooden block. The algorithm has less trouble reconstructing non-overlapping sources. The reconstruction errors are comparable to the ones of Experiment 1.

The setup of *Experiment* 3 is challenging, as the hot background introduces ghost accumulations that influence the reconstruction significantly. The error is smaller if both sources are comparable in their activities and position. Nevertheless, the results are less conclusive, as can be seen by the large error distribution for Positions 2 & 3, with two activity combinations of 30 MBq and 60 MBq

The results of *Experiment* 4 are mainly influenced by two factors: (1) the quality of the SLNF reconstruction and (2) the inside-out tracking. To assess these factors, the ground truth object (green cluster in Figs. 5, 6, and 7) was placed in the AR scene such that it is on top of the active source instead of being anchored on $T_3$ (*cf. *Fig. 3). This minimized potential inside-out tracking errors and mitigated a potential drift of the AR objects during the measurements. The reconstruction (red cluster in Figs. 5, 6, and 7), with its origin on $T_3$, was compared against this ground truth object to gauge accuracy. These comparisons allowed us to estimate whether systematic errors influenced the augmentation. As the errors were all in a similar range, namely from 0 mm to 4.47 mm, we assumed that no further inherent errors, *e.g.*, drift, etc., were significant. The speed of the computation mainly depends on the runtime of the SLNF algorithm and the settings of the $\gamma$-detector (*i.e.*, number of measurements, exposure time). Depending on the source activity, these settings can be adapted. And each measurement can be manually triggered by the surgeon. It is assumed that no quick motions around the region of interest, *i.e.*, the neck of the patient, occur during the biopsy. This helps to avoid drift. Further, the tracer distribution in the lymphatic system is a rather slow process. As such, the HL2 AR rendering, done at a frame rate of approx. 16 fps, is sufficient, thanks also to the stable inside-out tracking. We note that the differences between Experiments 1 and 4 are due to the physical nature of these experiments.

Neighboring lymph nodes might have different drainage paths and need thus be identified accordingly. As these nodules have a diameter between 5 mm and 10 mm, an upper error boundary should be at around 5 mm. This goal is met for single-source reconstructions. Two sources cannot be separated, given this boundary.

## Conclusion

This proof-of-concept study showed how an integrated approach works by combining a stationary $\gamma$-detector, a head-mounted display, and a reconstruction algorithm. Such a setup could support and improve sentinel lymph node biopsy (SNB) and thus also improve the patient’s outcome. The strenuous process of locating relevant lymph nodes using a hand-held $\gamma$-detecting probe can thus be significantly simplified using the power of AR. The detection and reconstruction of single sources with activities between 5 MBq and 60 MBq are robust, with errors in an acceptable range. The actual augmentation depends not only on the reconstruction quality but also on the inside-out tracking capabilities of the head-mounted display; in our case, it shows good accuracy and stability. The detection of multiple sources is challenging for the reconstruction algorithm. Thus, more experiments need to be done with phantoms, as well as with patient cohorts. Clinical measurements showed that the accumulated tracer in biologically active lymph nodes varies and is, in general, very low, *i.e.*, 0.1 % − 10 % of the initially injected volume [15]. Therefore, exposure time and the $\gamma$-detector placement are crucial for reliable data acquisition. Our experiments show promising results for the proposed approach and deserve further research.

## A Appendix

This Appendix aims to explain why we settled for the design of our collimator, and how we assessed the quality of the reconstruction of the source at different positions. To do so, we used an Optimal Design algorithm that was introduced in [13], which the authors in [8] applied to find a pinhole pattern for their collimator. We briefly recap of how the authors in [8] used the algorithm. Then we show that our new collimator is superior to theirs, and, in a final step, we explain how we can assess the performance of the collimator for different positions.

**Table 6:** Comparing the collimators of [8] with our collimator (random-hole collimator) on 100 randomly chosen subspaces with one, two, three, and four sources

Number of sources	1	2	3	4
${\mathcal {V}}({\textbf{1}})$ symmetric collimator w/ septum walls	5.5467	11.9921	17.0688	22.9842
${\mathcal {V}}({\textbf{1}})$ symmetric collimator w/o septum walls	1.0940	2.5263	3.4999	4.6190
${\mathcal {V}}({\textbf{1}})$ non-symmetric collimator SLNF	1.0326	2.4797	3.4194	4.5105
${\mathcal {V}}({\textbf{1}})$ random-hole collimator	1.0034	2.4614	3.3377	4.3964

### A.1 Background

In the approach of [8], they assumed $N_p=200$ possible pinholes and tried to find the optimal pinholes for a given subspace of *u*. To this end, they defined

4 $$\begin{aligned} {\mathcal {A}} = \begin{pmatrix} A_1 \\ \vdots \\ A_{N_p} \\ \end{pmatrix} \end{aligned}$$

where $A_n$ projects the subspace *u* on the detector, through the *n*th pinhole. We note that $A_n$ takes the nonzero values of *u* into account and omits the others. They find the pinholes which are good for the reconstruction of the nonzero entries of *u* by minimizing over the weights $w_m$ of the pinholes of the function

5 $$\begin{aligned} \mathop {\mathrm {arg\,min}}\limits _w \, \left( \sigma ^2{\mathcal {V}}(w) + \beta \sum w_m\right) \, \end{aligned}$$

where

6 $$\begin{aligned} {\mathcal {V}}(w) = \text {trace}\left( {\mathcal {W}}{\mathcal {A}}{\mathcal {C}}(w)^{-2}{\mathcal {A}}^T{\mathcal {W}}\right) \end{aligned}$$

with

7 $$\begin{aligned} {\mathcal {C}}(w)={\mathcal {A}}^T{\mathcal {W}}{\mathcal {A}} \quad \text {and} \quad {\mathcal {W}} = \begin{pmatrix} W_1 &{} &{} \\ &{} \ddots &{} \\ &{} &{} W_{N_p} \\ \end{pmatrix} \end{aligned}$$

for $W_m=\text {diag}(w_m,\dots ,w_m)\in {\mathbb {R}}^{N\times N}$ (with *N* being the number of pixels of the detector), and a specific $\beta$ and $\sigma$. In the final step, they chose the 24 pinholes with the highest weight $w_m$ for their collimator.

### A.2 Collimator

In the next step, we would like to improve the design of the collimator in comparison to the design of [8]. Similar to their approach, we compared four collimators with 24 pinholes. The first collimator had a regular pattern of 24 pinholes ($3 \times 8)$, where each pinhole separated by a septum wall (symmetric collimator w/ septum walls), the second collimator had its septum walls removed (symmetric collimator w/o septum walls), the third one was the design of [8] (non-symmetric collimator SLNF), and the final collimator was our design using random pinholes (random-hole collimator). We computed for each of the collimators the value of Eq. 6, with $w={\textbf{1}}=(1,\cdots ,1$) and $N_p = 24$, for 100 randomly-chosen subspaces (with one, two, three, or four sources of diameter 5 mm). We see in Table 6 that the mean value from ${\mathcal {V}}({\textbf{1}})$ is the lowest for our collimator and, therefore, a better design than the one used in [8].

### A.3 Assessment of the source position

To compare the different positions of the sources for a specific collimator, we chose for all weights $w_m$; hence, we have ${\mathcal {W}}=\text {diag}(1,\dots ,1)=E$ and reformulate Eq. 6 to

8 $$\begin{aligned} \begin{array}{rl} {\mathcal {V}}({\textbf{1}}) = &{} \text {trace}\left( {\mathcal {W}}{\mathcal {A}}{\mathcal {C}}(w)^{-2}{\mathcal {A}}^T{\mathcal {W}}\right) \\ = &{} \text {trace}\left( E{\mathcal {A}}\left( {\mathcal {A}}^TE{\mathcal {A}}\right) ^{-2}{\mathcal {A}}^TE\right) \\ = &{} \text {trace}\left( {\mathcal {A}}\left( {\mathcal {A}}^T{\mathcal {A}}\right) ^{-2}{\mathcal {A}}^T\right) . \end{array} \end{aligned}$$

To compare the nine positions given in Fig. 4, and assess which positions will be challenging to reconstruct, we assumed that each is a point source. That being the case, all values of the subspace *u* are 0 except for one. We note that the column of *A* is a linear combination of $A_1,\cdots , A_{24}$, where $A_n$ projects the subspace *u* onto the detector through the *n*th pinhole. We can use the column of *A* corresponding with the nonzero value of *u*, which is the projection of *u* onto the detector, instead of $\mathcal {A}$. To further simplify our equation, we write for the sources $i=1,\dots ,9$ the vector $d^{(i)}$ corresponding to the detector image, which is one of the columns of the matrix *A*. To assess the performance of the algorithm for the reconstruction of the specific source, we replace $\mathcal {A}$ with each source separately with $d^{(i)}$. Therefore, we are left with

$$\begin{aligned} \tilde{\mathcal {V}}(i) = \text {trace}\left( d^{(i)}\left( d^{(i)T}d^{(i)}\right) ^{-2}d^{(i)T}\right) . \end{aligned}$$

for $$i=1,\dots ,9$$.

## Footnotes

1 https://www.surgiceye.com.

2 http://physics.nist.gov/PhysRefData/XrayMassCoef/ElemTab/z74.html.
